# Use of Dynamic Magnetic Resonance of the Urethra in Surgical Planning of Vesicourethral Anastomotic Stenosis Urethroplasty

**DOI:** 10.1590/S1677-5538.IBJU.2025.0264

**Published:** 2025-08-30

**Authors:** Gustavo Fiedler, André Guilherme Cavalcanti, Leonardo Kayat Bittencourt, Suzan Menasce Goldman

**Affiliations:** 1 Universidade Federal de São Paulo São Paulo SP Brasil Universidade Federal de São Paulo – UNIFESP, São Paulo, SP, Brasil; 2 Universidade Federal do Estado do Rio de Janeiro Rio de Janeiro RJ Brasil Universidade Federal do Estado do Rio de Janeiro – UNIRIO, Rio de Janeiro, RJ, Brasil; 3 University Hospitals Cleveland Medical Center Cleveland OH United States University Hospitals Cleveland Medical Center, Cleveland, OH, United States

**Keywords:** Magnetic Resonance Imaging, Urethra, Cystoscopy

## Abstract

**Objective:**

To evaluate the impact of dynamic Magnetic Resonance of the Urethra (d-MRU) on postoperative results and their agreement with intraoperative findings in patients with urethral stricture after radical prostatectomy.

**Methods:**

Forty-eight male patients (mean age 65.2 ± 8.1 years) with vesicourethral anastomotic stenosis (VUAS) after radical prostatectomy confirmed by cystoscopy were evaluated using dynamic MRU and cystourethrography (CUG). They were divided into two groups: d-MRU and CUG. Patients in the d-MRU group were evaluated using a new MRI protocol: urethral filling with lidocaine gel and distal urethral obstruction with sterile gas tourniquet; MR urethrography including axial T1-weighted images, coronal space, sagittal T2-weighted, axial T2-weighted, sagittal maximum intensity projection (MIP) with urographic effect, voiding sagittal MIP, and T1-weighted with fat saturation (T1 fat-sat) before and after gadolinium enhancement. Dynamic imaging acquisition with motion images was performed during voiding.

**Results:**

No significant difference in restenosis rates was observed between the D-MRI and UCG groups (5.6% vs. 16.7%, respectively; p = 0.261), but a significant difference in vascular preservation (94.4% vs. 63.3%, p=0.016). We found consistent dynamic MRU and intraoperative measurements of VUAS. Intraclass correlation coefficients showed satisfactory to excellent levels of agreement between the two imaging modalities and a strong correlation of dynamic MRU and intraoperative findings. Additionally, the Bland-Altman analysis revealed an agreement bias close to zero.

**Conclusions:**

Dynamic MRU is a safe and appropriate evaluation method that can provide guidance for surgical treatment planning in patients with VUAS after radical prostatectomy.

## INTRODUCTION

Prostate cancer is the most common solid tumor among Western men, and over 80% of patients with localized disease undergo definitive treatment, either with radiation therapy or radical prostatectomy. Although radical prostatectomy is a curative option, it is associated with complications such as urinary incontinence, erectile dysfunction, and vesicourethral anastomotic stenosis (VUAS) (1, 2). Although less common, VUAS presents a significant surgical challenge and adversely affects patients’ quality of life (3, 4).

VUAS results from fibrosis at the anastomotic site, leading to urethral stricture and symptoms such as weak urinary stream, hesitancy, and post-void dribbling (3, 5). Risk factors include obesity, diabetes, and smoking (4). While most cases develop within the first-year post-surgery, the long-term incidence may be underestimated due to inconsistent diagnostic practices. Population-based studies report that up to 19.3% of patients may develop urethral strictures over ten years, with incidence varying between open and robot-assisted procedures (5-7).

Accurate diagnostic assessment is critical for surgical planning. While uroflowmetry and ultrasound are useful initial tools, imaging modalities such as retrograde urethrography, voiding cystourethrography (VCUG), and magnetic resonance urethrography (MRU) are essential to characterize stenosis. MRU provides high-resolution, multiplanar visualization of periurethral tissues, enabling precise assessment of stricture length, location, and spongiofibrosis extent, which are crucial for operative strategy (3, 8-11).

Reconstructive strategies for VUAS depend on stricture location and length. Short segment stenoses above the sphincter may be approached abdominally or transpubically, whereas longer membranous strictures typically require perineal urethroplasty (12, 13). Successful surgery relies on a comprehensive understanding of complex pelvic anatomy and meticulous preoperative mapping of the urethral tract. MRU can serve as a valuable adjunct to optimize outcomes and reduce complications (9, 10, 14, 15).

Therefore, this study aimed to evaluate the impact of dynamic MRU (d-MRU) findings on surgical outcomes and intraoperative decision-making in patients undergoing posterior anastomotic urethroplasty for VUAS following radical prostatectomy. We also examined the concordance between d-MRU and intraoperative findings to validate its utility in surgical planning.

## METHODS

This study was approved by the Federal University of São Paulo research ethics committee (protocol number CAAE 32210020.50000.5505). Informed consent was waived as the study used anonymous patient data.

Patients undergoing urethroplasty as the treatment of choice for UVAS after radical prostatectomy were prospectively evaluated from January 2018 to June 2023. All d-MRU assessments were performed at an imaging diagnostic center (Diagnósticos da América S.A. group) in the city of Rio de Janeiro, southeastern Brazil.

Patients with VUAS failing prior endoscopic management or where conservative treatment was unfeasible (complete obstruction of the urethral lumen) were included in the study. We compared preoperative d-MRU findings (meatal and distal location and stenosis length and caliber) with intraoperative findings. All patients were followed up for a minimum of six months. Cystoscopy was used for post-operative follow-up to assess surgical outcomes, including urethral patency and/or restenosis, and the need for further intervention. Cystoscopy was performed in those with suspected recurrent urinary symptoms and at least once in all patients regardless of any symptoms during the six-month follow-up after surgery.

To evaluate the impact of dynamic MRU (d-MRU) findings on surgical outcomes we compared the outcomes of interest—rate of restenosis and vascular preservation (bulbourethral arteries) —in the d-MRU group and a control group. The control group comprised patients with VUAS undergoing surgery performed by the same surgeon during the same time period but evaluated using CUG. All patients in the CUG group were considered suitable for surgical intervention via perineal approach based on the degree of stenosis (at or below the lower edge of the obturator foramen).

### Inclusion criteria

Patients with VUAS after radical prostatectomy failing conservative treatments (balloon dilation or endoscopic procedures) with or without adjuvant radiation therapy; d-MRU as the sole imaging modality used for preoperative evaluation and surgical planning (CUG evaluation was never performed or performed well before recent endoscopic procedures); CUG showing stenosis treatable via a perineal approach based on radiographic criteria for those in the control group.

### Non-inclusion criteria

Patients who underwent previous urethroplasty surgery.

### Exclusion criteria

Patients with recurrent neoplastic disease.

### Magnetic resonance imaging of the urethra protocol and image acquisition

All MRI assessments were performed on the Siemens Magneton Sonata scanner 1.5 T/ 43 mT (Siemens Medical Systems; Erlangen, Germany). We followed the same protocol for all patients in the d-MRU group and it included: axial T1-weighted sequences; T2-weighted and sagittal 3D sequences; coronal T2- weighted sequences; sagittal maximum intensity projection images (MIP) images; and sagittal T1-weighted fat saturation (fat-sat) before and after intravenous administration of a gadolinium-based contrast agent. We took measurements of VUAS and assessed the extent of spongiofibrosis associated.

All patients were pre-administrated a 500-mL intravenous saline solution. The evaluator followed a standard procedure: genital asepsis; intraurethral infiltration with lidocaine gel; and gauze wrapping of the glans with slight traction of the penis. Urographic T2-weighted images were acquired at rest and during voiding. Images were captured at 1-2 second intervals following relaxation and opening out of the posterior urethra. Curved planar reformation is a technique used for reconstruction in vascular imaging protocols that can be easily applied to the curved male urethra allowing for the identification of strictures in longitudinal sections. Additional 3D reconstructions allowed us to create interactive image models for surgical approach planning. Contrast-enhanced images were obtained using a gadolinium-based contrast agent at a dose of 0.2 mL/kg. This protocol also allowed accurate assessment of the extent of spongiofibrosis.

### Statistical analysis

First, we conducted a descriptive analysis of all collected data, estimating mean, standard deviation (SD), and absolute (n) and relative (%) frequencies. We used the independent samples t-test for group comparisons after confirming normality with the Shapiro–Wilk test. When normality was not met, we applied the Mann-Whitney test for numerical variables and the chi-square test for categorical variables.

To evaluate differences in surgery success rates (restenosis and vascular preservation) between groups, we used a two-tailed Z-test for two proportions. A Bland-Altman analysis assessed agreement between MRU and intraoperative findings, estimating the intraclass correlation coefficient (κ) with 95% confidence intervals (CIs). Agreement was classified as poor (κ < 0.40), satisfactory (κ = 0.40–0.75), or excellent (κ > 0.75).

For mean comparisons, we used a two-sample t-test when normality was confirmed and the Wilcoxon test when it was not. All analyses were performed using R v. 4.3.1 with ggplot2 and blandr packages, with a significance level set at p ≤ 0.05.

## RESULTS

In this study, we evaluated 48 patients divided into two groups: d-MRU and CUG. The patients’ mean age was 65.2 ± 8.1 years. Table-1 shows a comparison of patient characteristics between the two groups.

**Table 1:** Comparative analysis of patient characteristics assessed by dynamic magnetic resonance of the urethra (d-MRU) and cystourethrography (CUG), including the correlation between d-MRU findings and intraoperative assessments of urethral stricture.

Study variables		d-MRU	CUG	Total	p-value
N		18	30	48	
Age (years), mean (SD)		66.8 (8.3)	64.3 (8.0)	65.2 (8.1)	0.293^a^
**Risk factors, n (%)**					0.986^b^
	None	11 (61.1)	19 (63.3)	30 (62.5)	
	Diabetes mellitus	5 (27.8)	8 (26.7)	13 (27.1)	
	Smoking	2 (11.1)	3 (10.0)	5 (10.4)	
	Prior endoscopic procedures (n), mean (SD)	2.3 (2.5)	2.5 (1.7)	2.4 (2.0)	0.235^c^
**Cystostomy, n (%)**					0.491^b^
	Yes	6 (33.3)	6 (20.0)	12 (25.0)	
	No	12 (66.7)	24 (80.0)	36 (75.0)	
**Prior radiation therapy**					0.090^b^
	Yes	10 (55.6)	8 (26.7)	18 (37.5)	
	No	8 (44.4)	22 (73.3)	30 (62.5)	
Follow-up (months), mean (SD)		36.1 (20.1)	26.9 (13.5)	30.4 (16.7)	0.063^a^
**Type of surgery, n (%)**					0.230^b^
	Anastomosis	13 (72.2)	27 (90.0)	40 (83.3)	
	Graft	5 (27.8)	3 (10.0)	8 (16.7)	
	Stenosis length (mm), mean (SD)	17.2 (9.3)	14.7 (6.2)	15.7 (7.5)	0.545^c^
**Vascular preservation, n (%)**					0.016^d^
	Yes	17 (94.4)	19 (63.3)	36 (75.0)	
	Partial	0 (0.0)	3 (10.0)	3 (6.2)	
	No	1 (5.6)	8 (26.7)	9 (18.8)	
**Restenosis, n (%)**					0.261^d^
	No	17 (94.4)	25 (83.3)	42 (89.5)	
	Yes	1 (5.6)	5 (16.7)	6 (12.5)	

Urethral stenosis measurements	d-MRU	Intraoperative	p-value	ICC (95% CI)	Strength	
n	18	18				
Meatal and distal location (mm), mean (SD)	175.9 (24.2)	173.6 (22.0)	0.239^e^	0.943 (0.851, 0.979)	Excellent	
Length (mm), mean (SD)	17.2 (9.3)	17.0 (8.9)	0.713^b^ ^f^	0.983 (0.954, 0.994)	Excellent	
Caliber (mm), mean (SD)	1.2 (1.2)	1.8 (2.3)	0.181^f^	0.608 (0.197, 0.837)	Satisfactory	

SD = standard deviation;

a = t-test for independent samples;

b = chi-square test;

c: Mann-Whitney test;

d = two-proportion Z-test; ICC = Intraclass Correlation Coefficient; 95% CI = 95% Confidence Interval; SD = Standard Deviation;

e = t-test for paired samples;

f = Wilcoxon Test

We found a surgery success rate of 94.4% in the d-MRU group with a significant difference in vascular preservation. Restenosis occurred in only one patient (5.6%) in the d-MRU group compared to five (16.7%) in the CUG group. However, we found no difference in the restenosis rate between the groups.

**Figure 1:**
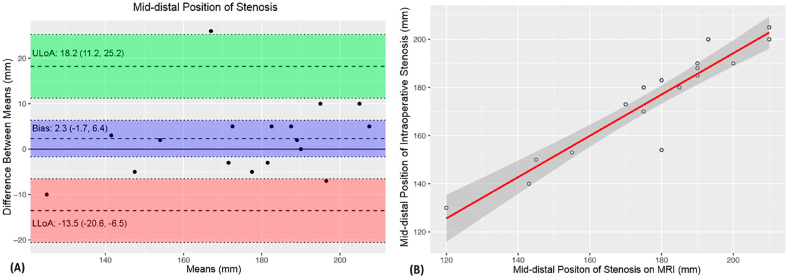
The Bland-Altman plot (A) shows a 2.3 mm agreement bias between meatal and distal urethral stenosis measurements. The scatter plot demonstrates a strong correlation between these measurements (B).

**Figure 2:**
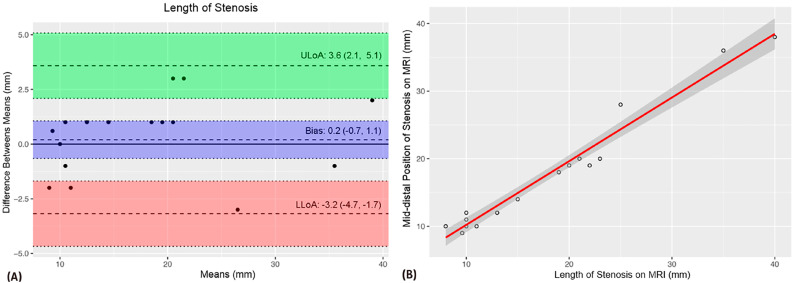
The Bland-Altman plot (A) shows a 0.2 mm agreement bias between urethral stenosis length measurements. The scatter plot demonstrates a strong correlation between these measurements (B).

**Figure 3:**
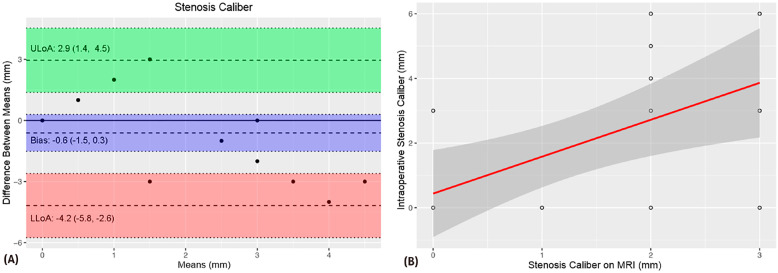
The Bland-Altman plot (A) shows a −0.6 mm agreement bias between urethral stenosis caliber measurements. The scatter plot demonstrates a satisfactory correlation between these measurements (B).

**Figure 4:**
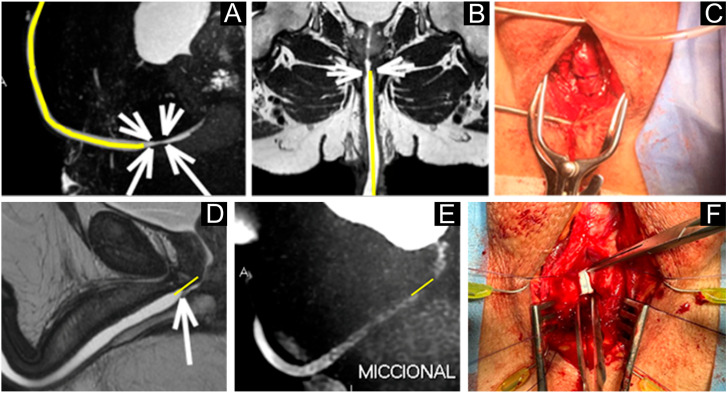
Dynamic magnetic resonance imaging of the urethra in different planes (A–B, D–E) showing the stenotic segment (arrows) and measurements of its length (yellow lines) and caliber. Intraoperative surgical views (C, F) confirm the location and extent of the stenosis, with findings consistent with the measurements obtained by magnetic resonance imaging.

**Figure 5:**
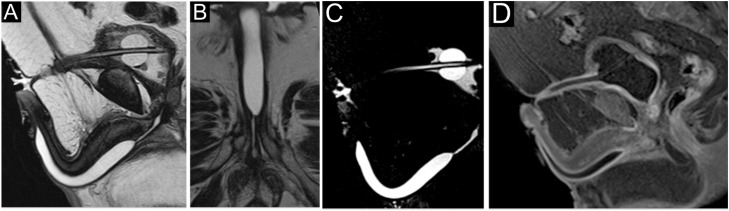
Seventy-year-old male patient with posterior urethral stenosis showing a ring-like appearance secondary to postoperative changes after radical prostatectomy. T2-weighted images (A–D) demonstrate a contrast-enhanced hypointense area consistent with spongiofibrosis at the stenotic segment.

A comparison of MRU and intraoperative measurements of VUAS (meatal and distal location, stenosis length and caliber) showed no significant difference of means (Table-1). The intraclass correlation coefficients (ICCs) revealed strength of agreement from satisfactory to excellent (Table-1). The Bland-Altman plots demonstrated agreement bias close to zero and a strong correlation between VUAS measurements (Figures 1-5).

## DISCUSSION

The present study is the first to evaluate the impact of d-MRU findings on surgical outcomes in patients with VUAS undergoing urethroplasty surgery after radical prostatectomy. It is the first study in the literature to exclusively focus on diagnostic assessment, management and follow-up of patients with non-traumatic posterior urethral stenosis. Our results showed no occurrence of restenosis in 94.4% in the d-MRU group versus 83.3% in the CUG group (p = 0.261).

The literature reports a VUAS rate of 1–8% whereas population studies have reported a 10-year cumulative rate of 19.3% (5). Short-segment anastomotic stenosis located above the urethral sphincter without prior radiation therapy can be reconstructed via the transabdominal and transpubic approach with a success rate of 60–83% and preservation of continence in about 70% of cases (12). Yet long-segment anastomotic stenosis within the membranous urethra require perineal urethral reconstruction with a success rate of approximately 80% (13). There are several open reconstruction approaches—abdominal, perineal, and combined abdominoperineal—, but a recurrent question is which is the best technique for urethroplasty. Surgical treatment failure rates may vary from 10-40% regardless of the surgical approach and the surgeon's experience level (3, 15). In our study, we found a surgical treatment failure rate of 5.6% in the d-MRU group versus 16.7% in the CUG group (p = 0.261).

Determining the exact location of the stenosis and resecting the entire diseased segment of the urethra are key for favorable surgery outcomes. However, so far, we have not been able to accurately predict its proximity to the striated urethral sphincter, exact length and the extent of surrounding fibrotic tissue. In addition, urethral and bladder neck mobilization during surgery should be performed to ensure tension-free anastomosis (16, 17). Few case series in the literature have examined traumatic posterior urethral stenoses, and they were mostly bulbar stenoses. Yet, in our study, we evaluated patients with VUAS after radical prostatectomy refractory to endoscopic treatment or with complete bladder neck obstruction. The sample included only those undergoing surgery via a perineal approach because it allows surgeons to navigate through a route of less fibrotic tissue minimizing the risk of injury to related structures. Our results unprecedentedly showed that d-MRU evaluation significantly increased vascular preservation compared to the CUG group (94.4% vs. 63.3%, p = 0.016).

The above-mentioned studies have reported consistent d-MRU and intraoperative measurements whereas stenosis length was likely overestimated with CUG evaluation (15, 18). In our sample, we also found consistent d-MRU and intraoperative measurements of VUAS. Intraclass correlation coefficients showed satisfactory to excellent strength of agreement between d-MRU and intraoperative measurements. Furthermore, there was a strong correlation between these measurements and an agreement bias close to zero using the Bland-Altman method.

Surgeons rely heavily on imaging data. In our study, the data obtained during voiding using a dynamic MRU protocol are very promising as they demonstrated a strong correlation between preoperative and intraoperative findings with no significant difference between measurements. There was an excellent strength of association between measurements of the distance from the stenosis to the urethral meatus and stenosis length, and satisfactory agreement of urethral caliber measurements.

MRI advances in the last two decades have allowed us to develop more effective protocols for urethral disease assessment. MRI offers the advantage of not exposing the patient to ionizing radiation and examinations can be performed in any MRI machine (19). The radiology team finds it an easily applicable technique and is included in most prostate evaluation protocols. It provides multiplanar high-resolution imaging with the estimation of density and depth of fibrotic tissue and allows visualization of the pelvis and surrounding perineal tissues. Two studies published in 2006 by Osman et al. (20). and Sung et al. (21). evaluated the use of MRU with new techniques and 1.5-T systems and showed its applicability for effectively diagnosing urethral diseases and providing additional data for surgical planning. Later, in 2010, Oh et al. (18). presented results evidencing more accurate assessments of the precise stenosis location when compared to CUG. They reported intraoperative changes in the surgical plan in 11 of 25 patients with traumatic posterior urethral stenosis (18). Tao et al. were the first to describe voiding MRU, a technique that is similar to VCUG and allows visualization of the urethra proximal to the stricture (15). In addition, MRU allows to predict the complexity of bulbar urethral stenoses through certain findings, including involvement of tunica albuginea, periurethral fistulas, extent of spongiofibrosis, and stenoses in the proximal and distal bulbar urethra (14).

MRU data can be reformatted into the correct image plane providing accurate assessments of stricture length and urethral lumen patency in millimeters. Three recent studies have reported improved MRU data available in urethral diseases (15, 22, 23). MRI has enjoyed significant growth in recent years and become a widely used diagnostic modality. MRI scans are available in non-specialized and specialized care centers. Current literature has reported a wide variety of MRU applications in posterior urethral stenoses that are not yet included in the AUA or EAU guidelines. Yet, our group has been working to optimize MRU protocols with dynamic views to provide additional relevant data for improving surgical planning and minimizing recurrent stenosis.

Parameters used in d-MRU provide new data on the real extent of urethral strictures/stenoses that can often be overestimated using conventional methods, including open bladder neck, posterior urethra mobility, conditions in surrounding anatomical structures associated with urethral stenosis disease, and overall patient-examiner interaction during voiding. Furthermore, the use of contrast agents allows us to evaluate delayed or abnormal enhancement and predict risk factors and complications. Advances in MRI protocols and image quality have improved diagnostic performance and ensured appropriate evaluation of urethral lesions and demonstrated significantly superior performance compared with other diagnostic modalities.

One limitation of our study is that we did not compare preoperative d-MRU measurements with CUG measurements. CUG has significant disadvantages that are well described in the literature as the bladder neck does not open during the voiding phase in contrast with d-MRU that allows to reliably assess spontaneous voiding.

Further studies are needed to investigate whether d-MRU findings may impact clinical decision-making in patients with posterior urethral stenosis after radical prostatectomy.

## CONCLUSIONS

Our findings showed higher rates of vascular preservation in patients evaluated by dynamic magnetic resonance of the urethra compared with cystourethrography. There was an excellent agreement between dynamic magnetic resonance of the urethra and intraoperative measurements of meatal and distal stenosis and urethral stenosis length and satisfactory agreement in stenosis caliber measurements.

## Data Availability

All data generated or analysed during this study are included in this published article

## ABBREVIATIONS

AUA: American Urological Association
EUA: European Urological Association
VUAS: Vesicourethral Anastomotic Stenosis
MRU: Magnetic Resonance Imaging of the Urethra
d-MRU: dynamic Magnetic Resonance of the Urethra
CUG: Cystourethrography
VCUG: Voiding Cystourethrography
MIP: Maximum Intensity Projection
